# The Prevalence, Indications, Outcomes of the Most Common Major Gynecological Surgeries in Kazakhstan and Recommendations for Potential Improvements into Public Health and Clinical Practice: Analysis of the National Electronic Healthcare System (2014–2019)

**DOI:** 10.3390/ijerph192214679

**Published:** 2022-11-09

**Authors:** Yesbolat Sakko, Gulzhanat Aimagambetova, Milan Terzic, Talshyn Ukybassova, Gauri Bapayeva, Arnur Gusmanov, Gulnur Zhakhina, Almira Zhantuyakova, Abduzhappar Gaipov

**Affiliations:** 1 Department of Medicine, School of Medicine, Nazarbayev University, Astana 010000, Kazakhstan; 2 Department of Biomedical Sciences, School of Medicine, Nazarbayev University, Astana 010000, Kazakhstan; 3 Clinical Academic Department of Women’s Health, CF “University Medical Center”, Astana 010000, Kazakhstan; 4 Department of Obstetrics, Gynecology and Reproductive Sciences, School of Medicine, University of Pittsburgh, 300 Halket Street, Pittsburgh, PA 15213, USA; 5 Department of Obstetrics and Gynecology, University of British Columbia, Vancouver, BC V6T 1Z4, Canada; 6 Clinical Academic Department of Internal Medicine, CF “University Medical Center”, Astana 010000, Kazakhstan

**Keywords:** hysterectomy, salpingectomy, oophorectomy, salpingo-oophorectomy, public health, Kazakhstan, epidemiology

## Abstract

Objectives: Major gynecological surgeries are indicated for the treatment of female genital pathologies. It is key to examine trends in gynecologic surgical procedures and updated recommendations by international gynecological societies to find opportunities for improvement of local guidelines. To date, a very limited number of reports have been published on the epidemiology of gynecological surgeries in Kazakhstan. Moreover, some local guidelines for gynecological conditions do not comply with the international recommendations. Thus, this study aims to investigate the prevalence, indications, and outcomes of the most common major gynecological surgeries by analyzing large-scale Kazakhstani healthcare data, and identifying possible opportunities for improvement of the local public health and clinical practice. Methods: A descriptive, population-based study among women who underwent a gynecological surgery in healthcare settings across the Republic of Kazakhstan during the period of 2014–2019 was performed. Data were collected from the Unified Nationwide Electronic Health System (UNEHS). Results: In total, 80,401 surgery cases were identified and analyzed in the UNEHS database for a period of 6 years (2014–2019). The median age of the participants was 40 years old, with 61.1% in reproductive age. The most prevalent intervention was a unilateral salpingectomy—29.4%, with 72.6% patients aged between 18–34 years. The proportion of different types of hysterectomies was 49.4%. In 20% of cases, subtotal abdominal hysterectomy was performed due to uterine leiomyoma. The proportion of laparoscopic procedures in Kazakhstani gynecological practice is as low—11.59%. Conclusions: The Kazakhstani public health and gynecological care sector should reinforce implementation of contemporary treatment methods and up-to-date policies and guidelines. The overall trends in surgical procedures performed for gynecological pathologies, including uterine leiomyoma and ectopic pregnancy treatment, should be changed in favor of the minimally invasive methods in order to adopt a fertility-sparing approach.

## 1. Introduction

Major gynecological surgeries (hysterectomy, salpingectomy, and oophorectomy) are common interventions in gynecological practice. These procedures are indicated for the treatment of female genital tract pathologies [1]. Hysterectomy is one of the most frequently performed major gynecological surgeries in women worldwide, and involves the removal of the uterus (whole or parts) [1,2,3,4,5,6]. It is estimated that 33% of women in the United States have had a hysterectomy by the age of 60. It is also the most common gynecological procedure in the United States, with more than 600,000 procedures performed annually [2,4,5,7]. Broadly, hysterectomy can be performed using three approaches: vaginal, laparoscopic, and abdominal approach depending on multiple factors and specific indications: patient’s age, uterine volume and mobility, body mass index, history of abdominal surgery, and nulliparity [2,6,8]. However, the study conducted by Aarts et al. (2015) shows that laparoscopic hysterectomy has advantages over abdominal hysterectomy, including more rapid recovery and fewer febrile episodes and wound or abdominal wall infections, but also a longer operating time. Laparoscopic and vaginal approaches have comparable safety profiles, with the vaginal approach taking less operative time [8].

The majority of hysterectomies are performed for benign conditions [2,4,5,6,9]. The most common indication is uterine leiomyoma, followed by abnormal uterine bleeding, pelvic masses, pelvic pain, and uterine prolapse [2,8,10,11]. The complication rate related to hysterectomy ranges from 2.3% to 19.2% depending on indications, risk factors, and the surgical approach utilized [1,12,13,14,15,16].

Salpingectomy is another common gynecological surgery that implies uterine tube removal [17]. It is indicated in cases of confirmed tubal ectopic pregnancy, tubo-ovarian abscess, and sactosalpinx as a preparation step for assisted reproductive technology (ART) treatment in patients with infertility. With new evidence about the tubal origin of ovarian cancer cells [18,19], many developed countries adopted an opportunistic salpingectomy as an intervention to prevent ovarian cancer [7,20,21,22,23,24,25].

Although various techniques are utilized for salpingectomy [17], this procedure is considered safe and is not associated with an increased rate of perioperative or postoperative complications [22]. Nevertheless, there are conflicting data regarding the impact of salpingectomy on anti-Mullerian hormone (AMH) levels: some studies found that the levels decreased after the surgery [22,26], while others report no impact of salpingectomy on AMH concentrations, ovarian reserve, or ovarian response [27,28].

Oophorectomy (ovariectomy), or removal of the ovaries, is commonly performed at the time of hysterectomy in order to treat various ovarian pathologies [7,25]. The most common indications for oophorectomy are unilateral/bilateral ovarian cysts or masses [7]. Some oophorectomy procedures are performed to prevent ovarian cancer in women who are at increased risk of ovarian cancer [21,25]. According to the studies, there is a clear survival benefit associated with prophylactic oophorectomy in patients with a family history of ovarian cancer and with mutations of BRCA1 and BRCA2 [25,29]. However, for average-risk women, the cancer risk reduction must be balanced with the complications and consequences of oophorectomy and imbalance in the sex hormones production [25]. In many cases, it is performed together with the removal of the fallopian tubes—salpingo-oophorectomy (salpingo-ovariectomy) [7,25,29]. According to the study by Jacoby et al. (2009), 63% of 461,321 women aged 45–49 years who underwent a hysterectomy in 2005 reported bilateral salpingo-oophorectomy (BSO) [7,10]. The most common indications for BSO are tubo-ovarian abscesses, pelvic inflammatory disease, and endometriosis [7].

Kazakhstan is a Central Asian state, rated as an upper-middle-income country based on the World Bank (WB) classification [30,31]. The country’s population comprises 19 million people, with the female population accounting for 52%, and the median age of women is 31.9 years [32]. The country’s healthcare system underwent profound changes after achieving independence in 1991. Starting from 2010, the financial resources of the healthcare system cover free medical care at the national level within the framework of the Unified National Health System (UNHS). Kazakhstan has also begun to promote evidence-based medicine approaches, develop and introduce new clinical guidelines, and implement processes to improve the quality of medical services [31,33]. The Unified Nationwide Electronic Health System (UNEHS) was introduced at the end of 2013 to integrate the healthcare data at the national level [31]. However, despite the recent development, many aspects of the healthcare system’s performance, including the availability of statistical and epidemiological data, require improvement. In the available published sources, there is no information on statistical data to assess the rates of the most commonly performed major gynecological surgeries and their outcomes in Kazakhstani healthcare settings. A very limited number of reports have been published on the rates of gynecological surgeries in Kazakhstan [31,34]. Moreover, some local guidelines for gynecological conditions do not comply with the international recommendations. Thus, the current study aims to investigate the prevalence, indications, and outcomes of the most common major gynecological surgeries (hysterectomy, salpingectomy, and oophorectomy) by analyzing large-scale Kazakhstani healthcare data from the national registry. Analysis of the database could help to identify possible opportunities for improvement of the local public health and gynecological practice.

## 2. Materials and Methods

### 2.1. Study Population and Data Sources

The study population included hospitalized patients who had any type of major gynecological surgery (hysterectomy, salpingectomy, or oophorectomy) performed in any hospital setting in Kazakhstan between 2014 and 2019. The information was obtained from the “Electronic Registry of Inpatients”—one of the components of the UNEHS, launched in late 2013 to consolidate healthcare data storage across the country’s healthcare system. Patient demographics (age, sex, ethnicity, residency place), dates of hospital admission and discharge, International Classification of Diseases, 10th edition (ICD-10) codes (https://www.icd10data.com/, accessed on 1 August 2022) for the main diagnosis, comorbidities, complications, and type of admission were among the indicated factors/variables in the retrieved raw data.

### 2.2. Patients Selection and Definitions

**Figure 1:**
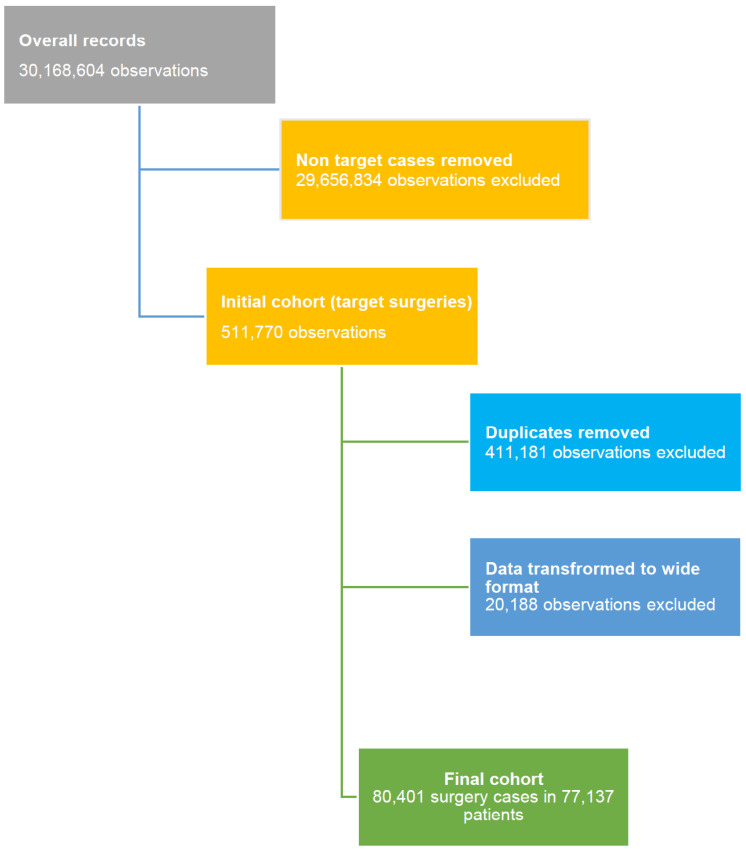
Patients’ selection flow chart.

Patients’ selection was carried out from the inpatient hospitals’ database of the UNEHS, searching surgical cases among 30,168,604 medical records according to the International Classification of Diseases, 9th edition (ICD-9) procedural codes (https://www.icd10data.com/, accessed on 1 August 2022). The following codes, registered in the UNEHS, were applied in order to retrieve information on the most common major gynecological surgeries—“65.3”, “65.51”, “65.53”, “65.61”, “65.62”, “66.4”, “66.51”, “66.62”, “68.3”, and “68.4”. These ICD-9 procedural codes were used as selection criteria for this study, yielding a total of 551,770 target surgeries. After removing duplicates and data cleaning, a dataset of 80,401 surgery cases was extracted, which are linked to 77,137 patients (Figure 1).

The extracted diagnoses were identified and categorized by the ICD-10 code, and the most prevalent diseases are provided in Supplementary Table S1. The records of diagnoses were originally labeled as the main diagnosis, comorbidities, or complications at hospitals based on their etiological–pathophysiological pathway.

### 2.3. Ethical Approval

The study was conducted in compliance with Helsinki declaration and approved by the Institutional Research Ethics Committee (IREC) of the Nazarbayev University, protocol NU-IREC 490/18112021, with exemption from informed consent due to the nature of the study.

### 2.4. Statistical Analysis

The descriptive analysis was performed in order to show the cohort’s demographic characteristics in frequencies and percentages. To assess the all-cause mortality hazard ratio (HR), crude and adjusted Cox regression modeling was performed, applying the Wald’s test for statistical significance. Cox regression models were adjusted for demographic factors and surgical procedures. Demographic categories with the largest number of patients were selected as reference groups. The ICD-9 code “68.3” (subtotal hysterectomy) performed for uterine leiomyoma was one of the most common indications for this type of surgery [1,4,6,9] was selected as the reference group for the surgical procedures’ variable in Cox regression analysis. The two-sided *p*-values reported as significant at <0.05 for every analysis. Data processing and statistical analysis were made using STATA 16 MP2 Version [35].

## 3. Results

### 3.1. Study Subjects Description

In total, 80,401 surgery cases, which are linked to 77,137 patients’ records, were identified and analyzed in the national electronic database for the period of 2014–2019, from all Kazakhstani regions. These patients underwent the most common gynecological surgeries (hysterectomy, salpingectomy, oophorectomy) due to specific indications. For the period of 6 years (2014–2019), 80,401 hysterectomy, salpingectomy, and oophorectomy surgeries were performed on 77,137 patients. As shown in Table 1, some patients had simultaneous procedures performed during the same surgical period, depending on the diagnosis and surgical indications: unilateral salpingo-oophorectomy, BSO, or combinations (simultaneous surgeries) of hysterectomy and BSO, etc. The most frequent combination was total abdominal hysterectomy with removal of both ovaries and tubes (Table 1).

A summary of the social and demographic characteristics of women is provided in Table 2. The participants’ ages ranged from 1 to 95 years, and the median age of the participants was 40 (IQR 31–49) years. The major proportions of the participants were in their reproductive age—34.2% of women aged between 18 and 34 years and 26.9% between 35 and 44 years. Only 0.4% of the study subjects (278 cases) were younger than 18 years old. The ethnic distribution of the investigated population includes 61.2% of Kazakh ethnicity, and 38.8% of other ethnic groups, including Russian. Patient death as an outcome was analyzed within two months after surgery and was linked to the particular hospitalization and procedure.

The distribution of cases analyzed from the regions of the country was almost equal; however, greater proportions were represented from the Almaty city (13.7%), the East Kazakhstan (13.1%), and Almaty (10.4%) regions, followed by the Karaganda region (9.2%). The proportion of the representatives from urban areas was 65.3% versus 34.8% of the rural population. Out of all analyzed records, 53.9% of patients passed through the emergency admission route due to urgent indications, and for 46.1% of patients, hospitalization was planned (elective). For the majority of patients, the outcome of treatment was positive, as they were discharged from hospitals either with a full recovery (62.9%) or with improvement (46.4%), (Table 2).

### 3.2. Analysis of the Surgical Procedures by Type, Indications, and Approach

Out of all 80,401 analyzed surgical procedures, the most common surgery was a unilateral salpingectomy (ICD-9 code “66.62”), performed in 29.4% of analyzed surgical cases (Table 1) indicated due to tubal ectopic pregnancy (Supplementary Table S2). The vast majority of these patients for whom a unilateral salpingectomy was performed were in their reproductive age, with 72.6% aged between 18–34 years and 26.9% between 35–44 years.

The proportion of different types of hysterectomies (ICD-9 codes “68.3”, “68.4”, and “68.61”) in the studied population for the period of 6 years (2014–2019) was 49.4%. Out of all hysterectomies, the most frequent procedure was subtotal abdominal hysterectomy (ICD-9 code “68.3”) performed in 20% due to uterine leiomyoma (Supplementary Table S2). In this group, most of the patients were of premenopausal age (45–50 years old)—34.9%, while 31.7% were in their late reproductive age (35–44 years old); and 23.1% of women had a subtotal abdominal hysterectomy in menopause (after 51 years old). A total abdominal hysterectomy (ICD-9 code “68.4”) was the third most prevalent surgical procedure among the studied population—18.28%, with 45.1% of patients being in their menopausal age at the time of surgery. The laparoscopic radical hysterectomy (ICD-9 code “68.61”) made up 11.13% of all surgical procedures analyzed. Similarly to the total abdominal hysterectomy procedure, laparoscopic radical hysterectomy was the most prevalent in the older age group.

Bilateral salpingectomy (ICD-9 code “66.51”) was performed in 3478 cases (4.3% of all procedures). In 630 patients (18%), it was performed together with hysterectomy for uterine leiomyoma (D.25), but for 138 (4%) patients, it was performed due to tubal ectopic pregnancy (Supplementary Table S2). Out of all cases of bilateral salpingectomy, 83.6% of patients were of reproductive age (18–35 years old), and only 16.4% were patients in the older groups (45–50 and >51). BSO (ICD-9 code “65.61”) was also more prevalent among the older age groups (45–50 and >51): 23.3% and 46.1%, respectively.

According to the analyzed database, the minimally invasive approach (laparoscopic surgery) was used in only 11.59% of cases (“68.61”, laparoscopic radical hysterectomies in 11.13%, and “65.53”, laparoscopic removal of ovaries in 0.46%) (Table 2), with the larger proportions performed in the urban facility: 72.2% and 81%, respectively, for planned surgical procedures. There are no available data on the mode of salpingectomy (laparoscopic or abdominal).

**Figure 2:**
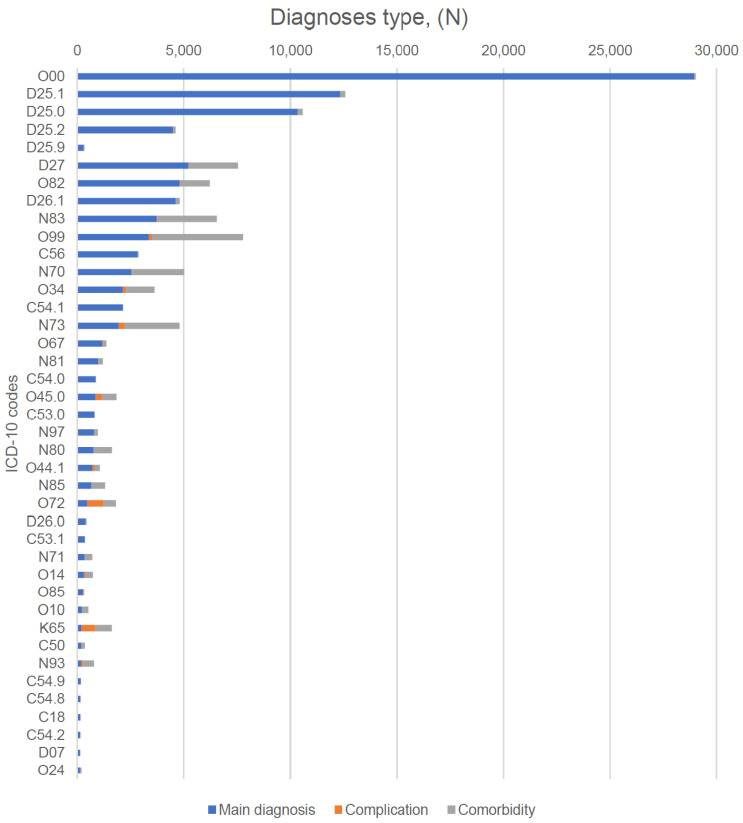
Indications for surgical procedures (top 40). Figure legend. ICD-10 codes: O00.1—ectopic pregnancy; D25.1—intramural leiomyoma of uterus; D25.0—sub-mucous leiomyoma of uterus; D25.2—subserosal leiomyoma of uterus; D25.9—leiomyoma of uterus, unspecified; D27—benign neoplasm of ovary; O82—single delivery by caesarean section; D26.1—other benign neoplasm of corpus uteri; N83—other assisted single delivery; O99—other maternal complications; C56—malignant neoplasm of ovary; N70—salpingitis and oophoritis; O34—maternal care of pelvic organs; C54.1—malignant neoplasm of endometrium; N73—other female pelvic inflammatory diseases; O67—labor and delivery complicated by intrapartum hemorrhage, not elsewhere classified; N81—female genital prolapse; C54.0—malignant neoplasm of isthmus uteri; O45.0—premature separation of placenta with coagulation defect; O53.0—malignant neoplasm of endocervix; N97—female infertility; N80—endometriosis; O44.1—placenta previa with hemorrhage; N85—other non-inflammatory disorders of the uterus, except cervix; O72—postpartum hemorrhage; D26.0—other benign neoplasm of cervix uteri; C53.1—malignant neoplasm of exocervix; N71—inflammatory disease of uterus, except cervix; O14—pre-eclampsia; O85—puerperal sepsis; O10—pre-existing hypertension complicating pregnancy, childbirth, and the puerperium; K65—peritonitis; C50—malignant neoplasm of breast; N93—other abnormal uterine and vaginal bleeding; C54.9—malignant neoplasm of corpus uteri, unspecified; C54.8—malignant neoplasm of overlapping sites of corpus uteri; C18—malignant neoplasm of colon; C54.2—malignant neoplasm of myometrium; D07—carcinoma in situ of other and unspecified genital organs; O24—diabetes mellitus in pregnancy, childbirth, and the puerperium.

Indications for the surgical procedures are presented in Figure 2 and Supplementary Table S3. As was mentioned above, the most prevalent indication for the surgical procedure was an ectopic pregnancy, or uterine leiomyoma of different localization. However, the indications were different among the 278 patients (Table 2) younger than 18 years old, consisting of: non-inflammatory disorders of the ovary (“N83”—28.25%), benign neoplasm of the ovary (“D27”—11.34%), and congenital malformation of the ovary (“Q50.39”—2.42%) (Figure 2).

The proportion of the malignant conditions reported in the general database (ICD-10: C56—malignant neoplasm of the ovary; C53.1—malignant neoplasm of exocervix; C54.9—malignant neoplasm of corpus uteri, unspecified; and other gynecological malignancy) made up a small proportion of the studied population (Supplementary Table S3).

**Figure 3:**
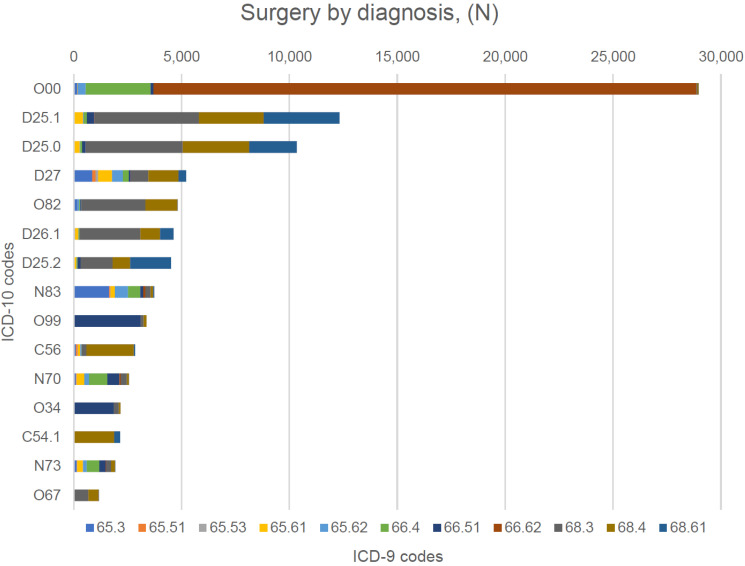
Types of the surgical procedures linked to the ICD-10 codes (top 15). Figure legend. ICD-9 codes: 65.3—unilateral oophorectomy; 65.51—other removal of both ovaries at the same operative episode; 65.53—laparoscopic removal of both ovaries at the same operative episode; 65.61—other removal of both ovaries and tubes at the same operative episode; 65.62—other removal of remaining ovary and tube; 66.4—total unilateral salpingectomy; 66.51—removal of both tubes; 66.62—salpingectomy with removal of tubal pregnancy; 68.3—subtotal abdominal hysterectomy; 68.4—total abdominal hysterectomy; 68.61—laparoscopic radical abdominal hysterectomy. ICD-10 codes: O00.1—ectopic pregnancy; D25.1—intramural leiomyoma of uterus; D25.0—submucous leiomyoma of uterus; D27—benign neoplasm of ovary; O82—single delivery by caesarean section; D26.1—other benign neoplasm of corpus uteri; D25.2—subserosal leiomyoma of uterus; N83—other assisted single delivery; O99—other maternal complications; C56—malignant neoplasm of ovary; N70—salpingitis and oophoritis; O34—maternal care of pelvic organs; C54.1—malignant neoplasm of endometrium; N73—other female pelvic inflammatory diseases; O67—labor and delivery complicated by intrapartum hemorrhage, not elsewhere classified.

The types of surgical procedures performed for the study subjects linked to the ICD-10 codes are presented on Figure 3.

### 3.3. Mortality Rates among the Study Population and Cox Regression Analysis of the Mortality

The mortality rates among the study subjects were the highest in the age group of 51 and older (161 patients—0.98%) (Table 3), as well as among the patients who had undergone the procedure for removal of both ovaries and total abdominal hysterectomy (ICD-9 code “68.4”) at the same operative episode (ICD9 code “65.51”)—2.91% and 1%, respectively. When the social determinants were linked to the surgery outcomes, the rates of mortality were seen to the highest among unemployed and retired patients (0.36% and 1.7%, respectively) (Table 3). In addition, the mortality rate was found to be the highest among the East Kazakhstan region and Almaty city residents—0.54% and 0.37%, respectively.

The Cox regression model includes survival HR, crude as well as adjusted for age, ethnicity, residence place, region, admission type, and surgical procedure. Patients in the age group older than 51 had the highest survival HR, among other age groups (Table 4).

Although showing crude HR differences, when adjusted to other variables, ethnicity did not show an effect on survival. Living in any region other than the capital city was shown to have a protective effect on survival, except for the Mangystau region. The highest survival was demonstrated in the country’s west—the Aktobe, Atyrau, and West Kazakhstan regions. On the other hand, Mangystau, also a western region, had one of the lowest survival rates in the country. Emergency admission was associated with high mortality, compared to planned admission. There was a significant association between survival HR and the surgery which the patient underwent. When compared to subtotal abdominal hysterectomy (ICD-9 code “68.3”), “65.51” (other removal of both ovaries at the same operative episode) + “65.53” (laparoscopic removal of both ovaries at the same operative episode), “65.61” (other removal of both ovaries and tubes at the same operative episode), and “68.4” (total unilateral salpingectomy) showed survival probabilities nearly two times lower. Adjusted HR values were 2.69, 2.12, and 1.87, respectively. The least lethal surgery was ICD-9 code “66.62” (salpingectomy with the removal of a tubal pregnancy).

## 4. Discussion

### 4.1. Public Health Implications for Healthcare Improvements in Kazakhstan

The Kazakhstani government prioritizes healthcare sector financial support [36]. The main directions of healthcare sector development are primary healthcare, improvement of the public health administration system, enhancing mother and child health services, and patients’ rehabilitation [36]. However, the country is rated by the WB as an upper-middle-income country, and the healthcare system’s financial support is within the scope of the governmental programs. In 2020, Kazakhstan’s healthcare sector received only 2.9% of the overall gross domestic product (GDP) [36]. Furthermore, although the Kazakhstani healthcare system has been improving the quality of maternal and gynecological care by implementing evidence-based national guidelines [33], there are still many blank spots and missing clinical care algorithms in this area. In particular, there is no national guideline for opportunistic salpingectomy as a prevention strategy for epithelial ovarian cancer. Therefore, in this study, our goal was to investigate the prevalence, indications, and outcomes of the most common major gynecological surgeries (hysterectomy, salpingectomy, and oophorectomy) in Kazakhstan. Analysis of the major gynecological surgeries in the country could be the first step in the development of national guidelines for opportunistic salpingectomy as a part of the ovarian cancer preventative measures, which have not yet been introduced in the country.

### 4.2. Outcomes of the Gynecological Surgeries in Kazakhstan and Recommendations for Potential Improvements

In total, 80,401 most common major gynecological surgery cases, as well as the available socio-demographic and clinical data, were analyzed in this study. As was seen from the database, out of all procedures analyzed within the studied 6-year period (2014–2019), the most common surgery was a unilateral salpingectomy (29.4%) performed in order to manage tubal ectopic pregnancy in reproductive-age patients. Bilateral salpingectomy made up a smaller proportion; however, in 83.6%, it was also performed in reproductive-age females. Such a huge proportion of unilateral and bilateral salpingectomies in reproductive-age women suggests a high rate of pelvic inflammatory diseases (PID) as one of the main risk factors of ectopic pregnancy [34,37]. Moreover, both PID and salpingectomy, especially bilateral, lead to infertility, thus increasing the demand for in vitro fertilization (IVF) in Kazakhstan [38,39]. Although some studies have found that unilateral salpingectomy for ectopic pregnancy by itself does not impair the ovarian reserve and response during IVF stimulation [40,41], contradictory results were reported for bilateral surgery [27]. Moreover, psychological stress related to the loss of tubes and subsequent sterility might contribute to the psychological distress existing in patients undergoing IVF procedures [38,42].

The high proportion of salpingectomies in cases of ectopic pregnancy in Kazakhstan is a reflection of the unavailability of national guidelines for medical management of ectopic pregnancy with methotrexate. This evidence-based approach is well-accepted in many developed countries, including the USA and the UK, where the national guidelines are developed and introduced into the gynecological practice [43,44]. A study from the USA confirms significantly increased use of methotrexate for the management of ectopic pregnancy, from 14.5% in 2006 to 27.3% by 2015 [45]. In the cited study, among the 62,588 women, 49,090 women (78.4%) were treated surgically, and the remaining 13,498 women (21.6%) received methotrexate [45], while in Kazakhstan all women with confirmed ectopic pregnancy undergo surgical treatment. Moreover, as confirmed by Gingold et al. (2021), even if the initial medical management of ectopic pregnancy with methotrexate is unsuccessful and there is a need to convert to surgical management, there is no decrease in the salpingostomy success rate associated with the previous methotrexate treatment [46]. Thus, there is an emergent need to update and introduce into the Kazakhstani clinical gynecological practice a guideline for ectopic pregnancy management providing an option for medical management with methotrexate. This could help to reduce the rates of salpingectomy at reproductive age.

At the same time, bilateral salpingectomy, a surgical procedure, which potentially may work to prevent epithelial ovarian cancer, was performed in only 3478 patients (out of the studied population of 80,401), with 16.4% being of post-reproductive age (45–50 and >51). According to the Kazakhstani national guidelines for female surgical sterilization, the procedure is currently performed via tubal ligation, rather than salpingectomy. However, in developed countries that have accepted an opportunistic salpingectomy as a preventative measure for ovarian cancer, rates of salpingectomies for permanent contraception are steadily increasing [47,48]. As reported by researchers, in the USA, 14.8% of subjects underwent a salpingectomy for permanent contraception within a 5-year period (2013–2017) [47]. According to these studies, laparoscopic bilateral salpingectomy is found to be a safe method for sterilization [23,48]. It does not increase perioperative risk compared with conventional tubal ligation [48], and may be preferred, where appropriate, to reduce the risk of ovarian cancer [23].

As the data in this study show, a large proportion of women underwent a subtotal or total hysterectomy (49.4%) within the studied 6-year period. Moreover, out of all these procedures, 43% of subtotal hysterectomies, 29.4% of total hysterectomies, and 23% of laparoscopic radical abdominal hysterectomies were performed in the 18–44 age group (reproductive age). Most of these procedures were performed for the management of uterine leiomyomas. This demonstrates the necessity to increase the minimally invasive approach for uterine leiomyoma treatment, such as uterine artery embolization (UAE), high intensity focused ultrasound (HIFU) [49], and transcervical fibroid ablation [50]. A similar South Korean study, which analyzed data collected over a 12-year period (2002–2013), found that the proportion of myomectomies increased by 2.24-fold (21% in 2002 to 47% in 2013), whereas the proportion of hysterectomies decreased by 0.62-fold (79% in 2002 to 49% in 2013) in their country [51]. Moreover, the researchers observed increased rates of the UAE and HIFU procedures. The study conducted by Lee et al. (2021) shows results, which are contrary to our findings, but also show very promising trends toward novel management in gynecological practice, which should be adopted in Kazakhstan.

While the proportion of minimally invasive procedures is increasing in developing and developed countries [47,51], the rate of laparoscopic procedures in Kazakhstani gynecological practice, according to the UNEHS database, is as low as 11.59%. The majority of them are performed in large cities. Thus, there is a need to improve the equipment supply to the healthcare system and provide appropriate physicians’ training.

In our study, the mortality rates were higher in emergency admissions than in planned/elective admissions. The mortality rates were the highest in patients who had undergone the procedure for removal of both ovaries and total abdominal hysterectomy at the same operative episode. Moreover, some regions had higher mortality rates—Almaty city, Almaty region, and the East Kazakhstan region. That could be explained by the overall number of procedures performed in these regions, which was higher than in the other regions—the more procedures performed, the higher the mortality rates. In this study, patients in the >51 age group had the highest survival hazard ratio in comparison with other age groups. This is comparable with the study where hysterectomy and BSO were not associated with poorer long-term survival when performed on women older than 45 years [52]. Unfortunately, there are no available data from other post-Soviet neighboring countries to compare the major gynecological surgeries’ prevalence/rates, indications, and complications.

### 4.3. Study Strengths and Limitations

Our study had several strengths. It is the first study that provides overall epidemiological data on the most common gynecological surgeries in Kazakhstan, including their prevalence, distribution, and mortality rates. Moreover, the analyzed cohort was large and covered the entire female population of Kazakhstan for the period of 6 years (2014–2019). Health records data were linked to the socio-demographic information; thus, it reduced potential misclassification and minimization of missing data. Nevertheless, some important limitations are associated with this report. These drawbacks are related to the UNEHS imperfections, which was introduced in 2014 and is still under development. The system does not provide detailed information on patients’ general past medical history, past pregnancy and delivery history, education, marital status, or family income. Availability of these important variables could enrich the study results. Moreover, in this study, we have not had an opportunity for a detailed analysis of the malignancies as indications for hysterectomies and BSO, as these data belong to the national oncology registry. Thus, it will be a task for our future investigations.

## 5. Conclusions

High-quality healthcare for women, especially in the reproductive period, has a positive impact on a country’s overall health indicators. The Kazakhstani public health gynecological care sector should reinforce implementation of contemporary treatment methods as well as up-to-date policies and guidelines. The overall trends in surgical procedures performed for gynecological pathologies, including uterine leiomyoma and ectopic pregnancy treatment, should be changed in favor of the minimally invasive methods in order to save the uterus and the patient’s fertility. National guidelines for opportunistic salpingectomy for epithelial ovarian cancer prevention should be developed and implemented in clinical practice. Incorporating laparoscopic bilateral salpingectomy as an option for female sterilization could contribute to epithelial ovarian cancer prevention after counseling patients regarding contraception and permanent sterilization.

## Figures and Tables

**Table 1:** Simultaneous surgical procedures incidence.

Procedure 1, (N)	Procedure 2, (N)												Total
	None	65.3	65.51	65.53	65.61	65.62	66.4	66.51	66.62	68.3	68.4	68.61	
65.3	3101	0	0	0	0	1	25	10	34	25	12	1	3209
65.51	322	0	0	0	0	0	1	1	0	5	2	0	331
65.53	307	0	0	0	0	1	0	6	0	2	0	33	349
65.61	1410	0	0	0	0	0	1	0	0	148	182	10	1751
65.62	1912	0	0	0	0	0	19	2	5	14	11	1	1964
66.4	5076	34	1	0	0	17	0	0	6	46	34	10	5224
66.51	3020	21	3	1	0	5	1	0	2	96	116	4	3269
66.62	23,530	22	0	0	0	5	13	8	0	2	0	0	23,580
68.3	15,376	23	6	5	176	18	36	75	4	0	16	2	15,737
68.4	13,978	8	2	0	187	7	30	98	0	9	0	1	14,320
68.61	8828	1	0	18	10	0	20	4	0	0	1	0	8882
Total	76,860	109	12	24	373	54	146	204	51	347	374	62	78,616

ICD-9 codes: 65.3—unilateral oophorectomy; 65.51—other removal of both ovaries at the same operative episode; 65.53—laparoscopic removal of both ovaries at the same operative episode; 65.61—other removal of both ovaries and tubes at the same operative episode; 65.62—other removal of remaining ovary and tube; 66.4—total unilateral salpingectomy; 66.51—removal of both tubes; 66.62—salpingectomy with removal of tubal pregnancy; 68.3—subtotal abdominal hysterectomy; 68.4—total abdominal hysterectomy; 68.61—laparoscopic radical abdominal hysterectomy.

**Table 2:** Socio-demographic characteristics of the study subjects.

Variables	Median (IQR) or N (%)	Procedures, N (%)										
		65.3	65.51	65.53	65.61	65.62	66.4	66.51	66.62	68.3	68.4	68.61
**Age**	40 (31; 49)											
**Age Group**												
<18	278 (0.4)	149 (4.5)	3 (0.9)	1 (0.3)	3 (0.1)	52 (2.6)	21 (0.4)	3 (0.1)	34 (0.1)	3 (0.0)	7 (0.1)	2 (0.0)
18–34	27,527 (34.2)	1545 (46.4)	61 (17.7)	15 (4.0)	186 (8.8)	827 (40.9)	3047 (56.7)	1470 (42.3)	17,153 (72.6)	1663 (10.3)	1437 (9.8)	123 (1.4)
35–44	21,600 (26.9)	928 (27.9)	66 (19.2)	34 (9.1)	462 (21.8)	671 (33.2)	1749 (32.6)	1437 (41.3)	6356 (26.9)	5092 (31.7)	2874 (19.6)	1931 (21.6)
45–50	14,627 (18.2)	345 (10.4)	52 (15.1)	43 (11.5)	494 (23.3)	233 (11.5)	396 (7.4)	427 (12.3)	83 (0.4)	5618 (34.9)	3759 (25.6)	3177 (35.5)
51+	16,369 (20.4)	360 (10.8)	162 (47.1)	280 (75.1)	979 (46.1)	237 (11.7)	159 (3.0)	141 (4.1)	6 (0.0)	3712 (23.1)	6621 (45.1)	3712 (41.5)
**Ethnicity**												
Kazakh	48,974 (61.2)	2162 (65.3)	200 (58.3)	157 (42.3)	1033 (48.8)	1254 (62.5)	3425 (64.1)	2242 (64.8)	15,950 (68.0)	9750 (60.9)	8216 (56.1)	4585 (51.4)
Other	12,309 (15.4)	504 (15.2)	61 (17.8)	72 (19.4)	315 (14.9)	290 (14.5)	775 (14.5)	563 (16.3)	3082 (13.1)	2524 (15.8)	2393 (16.3)	1730 (19.4)
Russian	18,709 (23.4)	644 (19.5)	82 (23.9)	142 (38.3)	770 (36.4)	461 (23.0)	1142 (21.4)	653 (18.9)	4441 (18.9)	3739 (23.4)	4037 (27.6)	2598 (29.2)
**Residence**												
Rural	27,942 (34.8)	1560 (46.9)	84 (24.4)	71 (19.0)	689 (32.4)	799 (39.6)	1900 (35.4)	1539 (44.3)	7939 (33.6)	6468 (40.2)	4402 (30.0)	2491 (27.9)
Urban	52,459 (65.3)	1767 (53.1)	260 (75.6)	302 (81.0)	1435 (67.6)	1221 (60.5)	3472 (64.6)	1939 (55.8)	15,693 (66.4)	9620 (59.8)	10,296 (70.1)	6454 (72.2)
**Region**												
Akmola region	2458 (3.1)	113 (3.4)	23 (6.7)	3 (0.8)	95 (4.5)	69 (3.4)	209 (3.9)	128 (3.7)	985 (4.2)	512 (3.2)	295 (2.0)	26 (0.3)
Aktobe region	3039 (3.8)	206 (6.2)	10 (2.9)	10 (2.7)	25 (1.2)	56 (2.8)	175 (3.3)	18 (0.5)	1197 (5.1)	922 (5.7)	331 (2.3)	89 (1.0)
Almaty city	11,048 (13.7)	180 (5.4)	16 (4.7)	16 (4.3)	67 (3.2)	91 (4.5)	468 (8.7)	1773 (51.0)	3049 (12.9)	595 (3.7)	2562 (17.4)	2231 (24.9)
Almaty region	8360 (10.4)	494 (14.9)	17 (4.9)	3 (0.8)	42 (2.0)	446 (22.1)	525 (9.8)	60 (1.7)	3726 (15.8)	2053 (12.8)	878 (6.0)	116 (1.3)
Astana (capital city)	5287 (6.6)	96 (2.9)	40 (11.6)	25 (6.7)	84 (4.0)	62 (3.1)	181 (3.4)	176 (5.1)	1319 (5.6)	776 (4.8)	848 (5.8)	1680 (18.8)
Atyrau region	1505 (1.9)	36 (1.1)	2 (0.6)	5 (1.3)	22 (1.0)	20 (1.0)	78 (1.5)	13 (0.4)	609 (2.6)	261 (1.6)	193 (1.3)	266 (3.0)
East Kazakhstan region	10,534 (13.1)	375 (11.3)	53 (15.4)	31 (8.3)	588 (27.7)	196 (9.7)	548 (10.2)	625 (18.0)	3277 (13.9)	2010 (12.5)	2184 (14.9)	647 (7.2)
Karaganda region	7425 (9.2)	309 (9.3)	18 (5.2)	223 (59.8)	400 (18.8)	229 (11.3)	593 (11.0)	205 (5.9)	1938 (8.2)	1774 (11.0)	1319 (9.0)	417 (4.7)
Kostanay region	4650 (5.8)	141 (4.2)	6 (1.7)	10 (2.7)	291 (13.7)	151 (7.5)	374 (7.0)	75 (2.2)	1305 (5.5)	1424 (8.9)	710 (4.8)	163 (1.8)
Kyzylorda region	2547 (3.2)	151 (4.5)	10 (2.9)	3 (0.8)	22 (1.0)	65 (3.2)	156 (2.9)	32 (0.9)	893 (3.8)	796 (5.0)	312 (2.1)	107 (1.2)
Mangystau region	669 (0.8)	16 (0.5)	0 (0.0)	3 (0.8)	1 (0.1)	10 (0.5)	29 (0.5)	7 (0.2)	94 (0.4)	116 (0.7)	54 (0.4)	339 (3.8)
North Kazakhstan region	2546 (3.2)	121 (3.6)	15 (4.4)	14 (3.8)	141 (6.6)	51 (2.5)	183 (3.4)	83 (2.4)	236 (1.0)	627 (3.9)	578 (3.9)	497 (5.6)
Pavlodar region	3885 (4.8)	127 (3.8)	16 (4.7)	8 (2.1)	59 (2.8)	108 (5.4)	197 (3.7)	71 (2.0)	635 (2.7)	1041 (6.5)	565 (3.8)	1058 (11.8)
Shymkent city	4207 (5.2)	126 (3.8)	78 (22.7)	1 (0.3)	21 (1.0)	22 (1.1)	615 (11.5)	12 (0.4)	1156 (4.9)	354 (2.2)	1003 (6.8)	819 (9.2)
Turkestan region	5247 (6.5)	491 (14.8)	17 (4.9)	4 (1.1)	21 (1.0)	243 (12.0)	407 (7.6)	32 (0.9)	1697 (7.2)	1383 (8.6)	805 (5.5)	147 (1.6)
West Kazakhstan region	2491 (3.1)	36 (1.1)	7 (2.0)	5 (1.3)	124 (5.8)	24 (1.2)	318 (5.9)	100 (2.9)	496 (2.1)	291 (1.8)	882 (6.0)	208 (2.3)
Zhambyl region	4503 (5.6)	309 (9.3)	16 (4.7)	9 (2.4)	121 (5.7)	177 (8.8)	316 (5.9)	68 (2.0)	1020 (4.3)	1153 (7.2)	1179 (8.0)	135 (1.5)
**Admission**												
Planned	37,066 (46.1)	1190 (35.8)	248 (72.1)	340 (91.2)	1179 (55.5)	627 (31.0)	1041 (19.4)	2307 (66.3)	259 (1.1)	10,825 (67.3)	11,094 (75.5)	7956 (88.9)
Emergency	43,335 (53.9)	2137 (64.2)	96 (27.9)	33 (8.9)	945 (44.5)	1393 (69.0)	4331 (80.6)	1171 (33.7)	23,373 (98.9)	5263 (32.7)	3604 (24.5)	989 (11.1)
**Outcome of stay**												
Discharge	79,603 (99.0)	3299 (99.2)	342 (99.4)	372 (99.7)	2090 (98.4)	1995 (98.8)	5325 (99.1)	3414 (98.2)	23,511 (99.5)	15,836 (98.4)	14,484 (98.5)	8935 (99.9)
Transfer	521 (0.7)	12 (0.4)	0 (0.0)	1 (0.3)	16 (0.8)	15 (0.7)	27 (0.5)	52 (1.5)	58 (0.3)	204 (1.3)	130 (0.9)	6 (0.1)
Voluntary discharge	104 (0.1)	5 (0.2)	0 (0.0)	0 (0.0)	0 (0.0)	5 (0.3)	12 (0.2)	4 (0.1)	63 (0.3)	6 (0.0)	9 (0.1)	0 (0.0)
Death	172 (0.2)	11 (0.3)	2 (0.6)	0 (0.0)	18 (0.9)	5 (0.3)	8 (0.2)	8 (0.2)	0 (0.0)	41 (0.3)	75 (0.5)	4 (0.0)
**Outcome of treatment**												
Without changes	343 (0.4)	6 (0.2)	12 (3.5)	0 (0.0)	4 (0.2)	4 (0.2)	6 (0.1)	2 (0.1)	16 (0.1)	102 (0.6)	191 (1.3)	0 (0.0)
Recovery	50,578 (62.9)	2140 (64.3)	156 (45.4)	301 (80.7)	1531 (72.1)	1431 (70.8)	3901 (72.6)	1378 (39.6)	14,668 (62.1)	11,694 (72.7)	7427 (50.5)	5951 (66.5)
Improvement	29,271 (36.4)	1166 (35.1)	173 (50.3)	72 (19.3)	571 (26.9)	580 (28.7)	1455 (27.1)	2088 (60.0)	8947 (37.9)	4242 (26.4)	6988 (47.6)	2,989 (33.4)
Deterioration	36 (0.0)	4 (0.1)	1 (0.3)	0 (0.0)	0 (0.0)	0 (0.0)	2 (0.0)	2 (0.1)	1 (0.0)	9 (0.1)	16 (0.1)	1 (0.0)
**Total, N (%)**	80,401 (100%)	3327 (4.14%)	344 (0.43%)	373 (0.46%)	2124 (2.64%)	2020 (2.51%)	5372 (6.68%)	3478 (4.33%)	23,632 (29.39%)	16,088 (20.01%)	14,698 (18.28%)	8945 (11.13%)

Outcome of stay terminology description: Discharge—patient went home after treatment; transfer—patient was transferred to another hospital; voluntary discharge—patient left a hospital before treatment was completed due to personal demand; death—patient death associated with treatment/surgery. Outcome of treatment terminology description: Without changes—patient was discharged without improvement; recovery—patient was discharged with recovery; improvement—patient was discharged with improvement; deterioration—patent was discharged/transferred to another hospital with deterioration.

**Table 3:** Mortality rates among the study subjects, linked to the socio-demographic determinants, and descriptive statistics.

Variables	Outcomes		
	Alive, N (%)	Deceased, N (%)	Total, N
**Age Group**			
<18	277 (99.64%)	1 (0.36%)	278
18–34	27,446 (99.71%)	81 (0.29%)	27,527
35–44	21,532 (99.69%)	68 (0.31%)	21,600
45–50	14,605 (99.85%)	22 (0.15%)	14,627
>51	16,208 (99.02%)	161 (0.98%)	16,369
**Social status**			
Disabled	528 (98.88%)	6 (1.12%)	534
Employed	33,309 (99.83%)	58 (0.17%)	33,367
Retiree	5682 (98.3%)	98 (1.7%)	5780
Unemployed	32,989 (99.64%)	120 (0.36%)	33,109
Other	7560 (99.33%)	51 (0.67%)	7611
**Region**			
Akmola region	2452 (99.76%)	6 (0.24%)	2458
Aktobe region	3035 (99.87%)	4 (0.13%)	3039
Almaty city	11,007 (99.63%)	41 (0.37%)	11,048
Almaty region	8322 (99.55%)	38 (0.45%)	8360
Astana (capital city)	5254 (99.38%)	33 (0.62%)	5287
Atyrau region	1503 (99.87%)	2 (0.13%)	1505
East Kazakhstan region	10,477 (99.46%)	57 (0.54%)	10,534
Karaganda region	7394 (99.58%)	31 (0.42%)	7425
Kostanay region	4636 (99.7%)	14 (0.3%)	4650
Kyzylorda region	2536 (99.57%)	11 (0.43%)	2547
Mangystau region	662 (98.95%)	7 (1.05%)	669
North Kazakhstan region	2530 (99.37%)	16 (0.63%)	2546
Pavlodar region	3866 (99.51%)	19 (0.49%)	3885
Shymkent city	4196 (99.74%)	11 (0.26%)	4207
Turkestan region	5226 (99.6%)	21 (0.4%)	5247
West Kazakhstan region	2483 (99.68%)	8 (0.32%)	2491
Zhambyl region	4489 (99.69%)	14 (0.31%)	4503
**Surgery (ICD-9 code)**			
Unilateral oophorectomy (65.3)	3308 (99.43%)	19 (0.57%)	3327
Other removal of both ovaries at the same operative episode (65.51)	334 (97.09%)	10 (2.91%)	344
Laparoscopic removal of both ovaries at the same operative episode (65.53)	373 (100%)	0 (0%)	373
Other removal of both ovaries and tubes at the same operative episode (65.61)	2095 (98.63%)	29 (1.37%)	2124
Other removal of remaining ovary and tube (65.62)	2011 (99.55%)	9 (0.45%)	2020
Total unilateral salpingectomy (66.4)	5359 (99.76%)	13 (0.24%)	5372
Removal of both tubes (66.51)	3467 (99.68%)	11 (0.32%)	3478
Salpingectomy with removal of tubal pregnancy (66.62)	23,630 (99.99%)	2 (0.01%)	23,632
Subtotal abdominal hysterectomy (68.3)	16,007 (99.5%)	81 (0.5%)	16,088
Total abdominal hysterectomy (68.4)	14,548 (98.98%)	150 (1.02%)	14,698
Laparoscopic radical abdominal hysterectomy (68.61)	8936 (99.9%)	9 (0.1%)	8945
**Total**	80,068 (99.59%)	333 (0.41%)	80,401

**Table 4:** Crude and adjusted Cox regression model with survival hazard ratio.

Variables	Crude HR	Crude *p*-Value	95% CI	Adjusted HR	Adjusted *p*-Value	95% CI
**Age group**						
<18	1.45	0.71	(0.20–10.45)	0.65	0.68	(0.09–4.79)
18–34	Ref.			Ref.		
35–44	1.05	0.79	(0.74–1.49)	0.71	0.07	(0.49–1.02)
45–50	0.55	0.02	(0.34–0.91)	0.31	0.00	(0.18–0.52)
51+	3.84	0.00	(2.89–5.11)	2.05	0.00	(1.44–2.92)
**Ethnicity**						
Kazakh	Ref.			Ref.		
Other	1.02	0.92	(0.73–1.42)	0.94	0.72	(0.67–1.32)
Russian	1.39	0.01	(1.07–1.79)	1.08	0.60	(0.82–1.43)
**Residence**						
Rural	0.86	0.21	(0.67–1.09)	0.89	0.40	(0.68–1.16)
Urban	Ref.			Ref.		
**Region**						
Akmola region	0.26	0.01	(0.09–0.74)	0.21	0.00	(0.08–0.61)
Aktobe region	0.21	0.00	(0.07–0.59)	0.19	0.00	(0.07–0.54)
Almaty city	0.57	0.02	(0.36–0.90)	0.52	0.01	(0.33–0.83)
Almaty region	0.61	0.05	(0.38–1.00)	0.66	0.11	(0.39–1.10)
Astana (capital city)	Ref.			Ref.		
Atyrau region	0.11	0.03	(0.01–0.78)	0.13	0.04	(0.02–0.93)
East Kazakhstan region	0.81	0.33	(0.52–1.25)	0.54	0.01	(0.34–0.85)
Karaganda region	0.58	0.04	(0.35–0.97)	0.39	0.00	(0.23–0.66)
Kostanay region	0.45	0.01	(0.24–0.85)	0.33	0.00	(0.17–0.63)
Kyzylorda region	0.63	0.20	(0.31–1.28)	0.66	0.25	(0.32–1.35)
Mangystau region	1.45	0.41	(0.61–3.45)	1.52	0.35	(0.63–3.67)
North Kazakhstan region	0.95	0.86	(0.51–1.74)	0.58	0.09	(0.31–1.09)
Pavlodar region	0.70	0.23	(0.39–1.26)	0.60	0.09	(0.33–1.09)
Shymkent city	0.38	0.01	(0.19–0.77)	0.33	0.00	(0.16–0.66)
Turkestan region	0.52	0.03	(0.29–0.93)	0.40	0.00	(0.22–0.74)
West Kazakhstan region	0.32	0.02	(0.13–0.82)	0.22	0.00	(0.09–0.58)
Zhambyl region	0.50	0.03	(0.27–0.93)	0.38	0.00	(0.20–0.71)
**Admission**						
Planned/elective	0.92	0.45	(0.73–1.15)	0.29	0.00	(0.22–0.37)
Urgent	Ref.			Ref.		
**Surgery (ICD-9)**						
Unilateral oophorectomy (65.3)	1.29	0.34	(0.77–2.16)	0.91	0.73	(0.53–1.56)
Other removal of both ovaries at the same operative episode (65.51)	3.33	0.00	(1.71–6.46)	2.69	0.00	(1.36–5.29)
Other removal of both ovaries and tubes at the same operative episode (65.61)	3.05	0.00	(1.95–4.76)	2.12	0.00	(1.33–3.37)
Other removal of remaining ovary and tube (65.62)	1.06	0.87	(0.53–2.13)	0.68	0.28	(0.34–1.38)
Total unilateral salpingectomy (66.4)	0.53	0.04	(0.29–0.98)	0.35	0.00	(0.18–0.66)
Removal of both tubes (66.51)	0.62	0.17	(0.31–1.23)	0.62	0.19	(0.30–1.27)
Salpingectomy with removal of tubal pregnancy (66.62)	0.02	0.00	(0.00–0.08)	0.01	0.00	(0.00–0.04)
Subtotal abdominal hysterectomy (68.3)	Ref.			Ref.		
Total abdominal hysterectomy (68.4)	2.19	0.00	(1.64–2.94)	1.87	0.00	(1.38–2.54)
Laparoscopic radical abdominal hysterectomy (68.61)	0.24	0.00	(0.12–0.48)	0.21	0.00	(0.10–0.43)

## Data Availability

All data related to this study are available from Republican Center for Electronic Health of the Ministry of Health of the Republic of Kazakhstan, but restrictions apply to the availability of these data, which were used under the contract-agreement for the current study, and so are not publicly available. Data are, however, available from the authors (abduzhappar.gaipov@nu.edu.kz) upon reasonable request and with permission of Ministry of Health of the Republic of Kazakhstan.

## Supplementary Materials

The following supporting information can be downloaded at: https://www.mdpi.com/article/10.3390/ijerph192214679/s1, Table S1: Outcome of stay in relation to the main diagnosis; Table S2: Surgical procedures link to the main diagnosis; Table S3: Most common diagnoses with complications and comorbidities.

## References

[1] Ortiz-Martínez R.A., Betancourt-Cañas A.J., Bolaños-Náñez D.M., Cardona-Narváez T., Portilla E.D., Flórez-Victoria O. (2018). Prevalence of surgical complications in gynecological surgery at the Hospital Universitario San José in Popayán, Colombia. 2015. Rev. Fac. Med..

[2] Ramdhan R.C., Loukas M., Tubbs R.S. (2017). Anatomical complications of hysterectomy: A review. Clin. Anat..

[3] Harnod T., Chen W., Wang J.-H., Lin S.-Z., Ding D.-C. (2018). Hysterectomies Are Associated with an Increased Risk of Depression: A Population-Based Cohort Study. J. Clin. Med..

[4] Orhan A., Ozerkan K., Kasapoglu I., Ocakoglu G., Demir B.C., Gunaydin T., Uncu G. (2019). Laparoscopic hysterectomy trends in challenging cases (1995–2018). J. Gynecol. Obstet. Hum. Reprod..

[5] Clarke-Pearson D.L., Geller E.J. (2013). Complications of hysterectomy. Obstet. Gynecol..

[6] Dedden S.J., Geomini P.M., Huirne J.A., Bongers M.Y. (2017). Vaginal and Laparoscopic hysterectomy as an outpatient procedure: A systematic review. Eur. J. Obstet. Gynecol. Reprod. Biol..

[7] Lozada Y., Bhagavath B. (2017). A Review of Laparoscopic Salpingo-Oophorectomy: Technique and Perioperative Considerations. J. Minim. Invasive Gynecol..

[8] Aarts J.W., E Nieboer T., Johnson N., Tavender E., Garry R., Mol B.W.J., Kluivers K.B. (2015). Surgical approach to hysterectomy for benign gynaecological disease. Cochrane Database Syst. Rev..

[9] Lycke K.D., Kahlert J., Damgaard R., Mogensen O., Hammer A. (2021). Trends in Hysterectomy Incidence Rates During 2000–2015 in Denmark: Shifting from Abdominal to Minimally Invasive Surgical Procedures. Clin. Epidemiol..

[10] Jacoby V.L., Autry A., Jacobson G., Domush R., Nakagawa S., Jacoby A. (2009). Nationwide Use of Laparoscopic Hysterectomy Compared With Abdominal and Vaginal Approaches. Obstet. Gynecol..

[11] Fortin C., Hur C., Falcone T. (2019). Impact of Laparoscopic Hysterectomy on Quality of Life. J. Minim. Invasive Gynecol..

[12] Brummer T.H., Jalkanen J., Fraser J., Heikkinen A.M., Kauko M., Mäkinen J., Seppälä T., Sjöberg J., Tomás E., Härkki P. (2011). FINHYST, a prospective study of 5279 hysterectomies: Complications and their risk factors. Hum. Reprod..

[13] McPherson K., Metcalfe M., Herbert A., Maresh M., Casbard A., Hargreaves J., Bridgman S., Clarke A. (2004). Severe complications of hysterectomy: The VALUE study. BJOG.

[14] English E.M., Bell S., Kamdar N.S., Swenson C.W., Wiese H., Morgan D.M. (2019). Importance of Estimated Blood Loss in Resource Utilization and Complications of Hysterectomy for Benign Indications. Obstet. Gynecol..

[15] Ellinides A., Manolopoulos P.P., Hajymiri M., Sergentanis T.N., Trompoukis P., Ntourakis D. (2022). Outpatient Hysterectomy versus Inpatient Hysterectomy: A Systematic Review and Meta-analysis. J. Minim. Invasive Gynecol..

[16] Uccella S., Zorzato P.C., Kho R.M. (2021). Incidence and Prevention of Vaginal Cuff Dehiscence after Laparoscopic and Robotic Hysterectomy: A Systematic Review and Meta-analysis. J. Minim. Invasive Gynecol..

[17] Ozcan M.C., Wilson J.R., Frishman G.N. (2021). A Systematic Review and Meta-analysis of Surgical Treatment of Ectopic Pregnancy with Salpingectomy versus Salpingostomy. J. Minim. Invasive Gynecol..

[18] Matulonis U.A., Sood A.K., Fallowfield L., Howitt B., Sehouli J., Karlan B.Y. (2016). Ovarian cancer. Nat. Rev. Dis. Prim..

[19] Bergsten T.M., Burdette J.E., Dean M. (2020). Fallopian tube initiation of high grade serous ovarian cancer and ovarian metastasis: Mechanisms and therapeutic implications. Cancer Lett..

[20] Dilley S., Straughn J.M., Leath C.A. (2017). The Evolution of and Evidence for Opportunistic Salpingectomy. Obstet. Gynecol..

[21] (2019). ACOG Committee Opinion No. 774: Opportunistic Salpingectomy as a Strategy for Epithelial Ovarian Cancer Prevention. Obstet. Gynecol..

[22] Kotsopoulos J., Narod S.A. (2020). Prophylactic salpingectomy for the prevention of ovarian cancer: Who should we target?. Int. J. Cancer.

[23] Mills K., Marchand G., Sainz K., Azadi A., Ware K., Vallejo J., Anderson S., King A., Osborn A., Ruther S. (2021). Salpingectomy vs tubal ligation for sterilization: A systematic review and meta-analysis. Am. J. Obstet. Gynecol..

[24] Kyo S., Ishikawa N., Nakamura K., Nakayama K. (2020). The fallopian tube as origin of ovarian cancer: Change of diagnostic and preventive strategies. Cancer Med..

[25] Evans E.C., Matteson K.A., Orejuela F.J., Alperin M., Balk E.M., El-Nashar S., Gleason J.L., Grimes C., Jeppson P., Mathews C. (2016). Salpingo-oophorectomy at the Time of Benign Hysterectomy: A Systematic Review. Obstet. Gynecol..

[26] Kotlyar A., Gingold J., Shue S., Falcone T. (2017). The Effect of Salpingectomy on Ovarian Function. J. Minim. Invasive Gynecol..

[27] Noventa M., Gizzo S., Saccardi C., Borgato S., Vitagliano A., Quaranta M., Litta P., Gangemi M., Ambrosini G., D’Antona D. (2016). Salpingectomy before assisted reproductive technologies: A systematic literature review. J. Ovarian Res..

[28] Luo J., Shi Y., Liu D., Yang D., Wu J., Cao L., Geng L., Hou Z., Lin H., Zhang Q. (2019). The effect of salpingectomy on the ovarian reserve and ovarian response in ectopic pregnancy: A systematic review and meta-analysis. Medicine.

[29] Eleje G.U., Eke A.C., Ezebialu I.U., Ikechebelu J.I., Ugwu E.O., Okonkwo O.O. (2018). Risk-reducing bilateral salpingo-oophorectomy in women with BRCA1 or BRCA2 mutations. Cochrane Database Syst. Rev..

[30] The World Bank Country Classification by Income. https://data.worldbank.org/country.

[31] Aimagambetova G., Sakko Y., Gusmanov A., Issanov A., Ukybassova T., Bapayeva G., Marat A., Nurpeissova A., Gaipov A. (2022). The Prevalence, Incidence, Indications and Outcomes of Peripartum Hysterectomy in Kazakhstan: Data from Unified Nationwide Electronic Healthcare System 2014–2018. Int. J. Womens Health.

[32] World Population Review Kazakhstan. https://worldpopulationreview.com/countries/kazakhstan-population.

[33] Kamalbekova G., Kalieva M. (2015). Evidence-based medicine Training: Kazakhstan experience. Int. J. Risk Saf. Med..

[34] Aketayeva A., Khamidullina Z., Akhmetova Z., Baubekova A., Khismetova Z., Dudnik Y., Aitbaeva Z. (2018). Diagnosis and Treatment of Female Infertility Is One of the Major Problems in Modern Gynecology. Iran. J. Public Health.

[35] StataCorp LLC (2019). Stata Statistical Software, Release 16.

[36] International Trade Administration Kazakhstan—Country Commercial Guide. https://www.trade.gov/country-commercial-guides/kazakhstan-healthcare.

[37] Ng K.Y.B., Cheong Y. (2019). Hydrosalpinx—Salpingostomy, salpingectomy or tubal occlusion. Best Pract. Res. Clin. Obstet. Gynaecol..

[38] Bapayeva G., Aimagambetova G., Issanov A., Terzic S., Ukybassova T., Aldiyarova A., Utepova G., Daribay Z., Bekbossinova G., Balykov A. (2021). The Effect of Stress, Anxiety and Depression on In Vitro Fertilization Outcome in Kazakhstani Public Clinical Setting: A Cross-Sectional Study. J. Clin. Med..

[39] Issanov A., Aimagambetova G., Terzic S., Bapayeva G., Ukybassova T., Baikoshkarova S., Utepova G., Daribay Z., Bekbossinova G., Balykov A. (2022). Impact of governmental support to the IVF clinical pregnancy rates: Differences between public and private clinical settings in Kazakhstan—A prospective cohort study. BMJ Open.

[40] Ödesjö E., Bergh C., Strandell A. (2015). Surgical methods for tubal pregnancy—Effects on ovarian response to controlled stimulation during IVF. Acta Obstet. Gynecol. Scand..

[41] Gay C., Perrin J., Courbiere B., Bretelle F., Agostini A. (2019). Impact of salpingectomy for ectopic pregnancy on the ovarian response during IVF stimulation. J. Gynecol. Obstet. Hum. Reprod..

[42] Aimagambetova G., Issanov A., Terzic S., Bapayeva G., Ukybassova T., Baikoshkarova S., Aldiyarova A., Shauyen F., Terzic M. (2020). The effect of psychological distress on IVF outcomes: Reality or speculations?. PLoS ONE.

[43] (2018). Committee on Practice Bulletins—Gynecology ACOG Practice Bulletin No. 191: Tubal Ectopic Pregnancy. Obstet. Gynecol..

[44] Elson C.J., Salim R., Potdar N., Chetty M., Ross J.A., Kirk E.J. (2016). on behalf of the Royal College of Obstetricians and Gynaecologists. Diagnosis and management of ectopic pregnancy. BJOG.

[45] Hsu J., Chen L., Gumer A.R., Tergas A.I., Hou J.Y., Burke W.M., Ananth C.V., Hershman D.L., Wright J.D. (2017). Disparities in the management of ectopic pregnancy. Am. J. Obstet. Gynecol..

[46] Gingold J.A., Janmey I., Gemmell L., Mei L., Falcone T. (2021). Effect of Methotrexate on Salpingostomy Completion Rate for Tubal Ectopic Pregnancy: A Retrospective Cohort Study. J. Minim. Invasive Gynecol..

[47] Polen-De C., Meganathan K., Lang P., Hohmann S., Jackson A., Whiteside J.L. (2019). Nationwide salpingectomy rates for an indication of permanent contraception before and after published practice guidelines. Contraception.

[48] Kim A.J., Barberio A., Berens P., Chen H.-Y., Gants S., Swilinski L., Acholonu U., Chang-Jackson S.-C. (2019). The Trend, Feasibility, and Safety of Salpingectomy as a form of Permanent Sterilization. J. Minim. Invasive Gynecol..

[49] Yerezhepbayeva M., Terzic M., Aimagambetova G., Crape B. (2022). Comparison of two invasive non-surgical treatment options for uterine myomas: Uterine artery embolization and magnetic resonance guided high intensity focused ultrasound—Systematic review. BMC Womens Health.

[50] Arnreiter C., Oppelt P. (2021). A Systematic Review of the Treatment of Uterine Myomas Using Transcervical Ultrasound-Guided Radiofrequency Ablation with the Sonata System. J. Minim. Invasive Gynecol..

[51] Lee M., Chung Y.-J., Kim H.-K., Hwang H., Park J.Y., Shin I., Kim C., Cho H.-H., Kim M., Jung C.Y. (2021). Estimated Prevalence and Incidence of Uterine Leiomyoma, and Its Treatment Trend in South Korean Women for 12 years: A National Population-Based Study. J. Women’s Health.

[52] Tuesley K.M., Protani M.M., Webb P.M., Dixon-Suen S.C., Wilson L.F., Stewart L.M., Jordan S.J. (2020). Hysterectomy with and without oophorectomy and all-cause and cause-specific mortality. Am. J. Obstet. Gynecol..

