# Supplementary material for: The Prevalence, Indications, Outcomes of the Most Common Major Gynecological Surgeries in Kazakhstan and Recommendations for Potential Improvements into Public Health and Clinical Practice: Analysis of the National Electronic Healthcare System (2014–2019)
Source: Int J Environ Res Public Health. 2022 Nov 9;19(22):14679. doi: 10.3390/ijerph192214679 (PMC9690357; doi:10.3390/ijerph192214679)
Supplement: Supplementary file 1 [file ijerph-19-14679-s001.zip › ijerph-1892194-supplementary.pdf]

Table S1. Outcome of stay in relation to the main diagnosis.

Diagnosis	ICD 10 code	Alive N (%)	Dead N (%)	Total
**Main Diagnosis**				
Ectopic pregnancy	O00	28,958 (99.99%)	4 (0.01%)	28,962
Intramural leiomyoma of uterus	D25.1	12,306 (99.85%)	18 (0.15%)	12,324
Submucous leiomyoma of uterus	D25.0	10,334 (99.88%)	12 (0.12%)	10,346
Benign neoplasm of ovary	D27	5199 (99.71%)	15 (0.29%)	5214
Single delivery by caesarean section	O82	4958 (99.52%)	24 (0.48%)	4982
Other benign neoplasm of corpus uteri	D26.1	4617 (99.78%)	10 (0.22%)	4627
Subserosal leiomyoma of uterus	D25.2	4510 (99.91%)	4 (0.09%)	4514
Other assisted single delivery	N83	3724 (99.65%)	13 (0.35%)	3737
Other maternal complications	O99	3339 (98.87%)	38 (1.13%)	3377
Malignant neoplasm of ovary	C56	2760 (96.98%)	86 (3.02%)	2846
Salpingitis and oophoritis	N70	2532 (98.75%)	32 (1.25%)	2564
Maternal care of pelvic organs	O34	2168 (100%)	0 (0%)	2168
Malignant neoplasm of endometrium	C54.1	2127 (98.56%)	31 (1.44%)	2158
Other female pelvic inflammatory diseases	N73	1896 (98.24%)	34 (1.76%)	1930
Female genital prolapse	N81	974 (99.9%)	1 (0.1%)	975
Malignant neoplasm of isthmus uteri	C54.0	844 (97.57%)	21 (2.43%)	865
Malignant neoplasm of endocervix	C53.0	800 (99.88%)	1 (0.12%)	801
Female infertility	N97	794 (100%)	0 (0%)	794
Endometriosis	N80	759 (100%)	0 (0%)	759
Single spontaneous delivery	O80	709 (99.72%)	2 (0.28%)	711
Total		104,216 (99.29%)	742 (0.71%)	104,958
**Comorbidity**				
Iron deficiency anemia	D50	7517 (99.71%)	22 (0.29%)	7539
Other maternal complications	O99	4280 (99.3%)	30 (0.7%)	4310
Other assisted single delivery	N83	2786 (99.82%)	5 (0.18%)	2791
Other female pelvic inflammatory diseases	N73	2587 (99.54%)	12 (0.46%)	2599
Salpingitis and oophoritis	N70	2430 (99.26%)	18 (0.74%)	2448
Benign neoplasm of ovary	D27	2318 (99.83%)	4 (0.17%)	2322
Essential (primary) hypertension	I10	1930 (98.87%)	22 (1.13%)	1952
Hypertensive heart disease	I11	1475 (98.6%)	21 (1.4%)	1496
Single delivery by caesarean section	O82	1388 (99.57%)	6 (0.43%)	1394

Maternal care of pelvic organs	O34	1336 (99.7%)	4 (0.3%)	1340
Obesity	E66	1264 (99.61%)	5 (0.39%)	1269
Supervision of high-risk pregnancy	Z35	938 (99.89%)	1 (0.11%)	939
Endometriosis	N80	865 (99.88%)	1 (0.12%)	866
Erosion and ectropion of cervix uteri	N86	835 (100%)	0 (0%)	835
Acute posthaemorrhagic anaemia	D62	785 (98.99%)	8 (1.01%)	793
Other disorders of peritoneum	K66	715 (99.58%)	3 (0.42%)	718
Other non-inflammatory disorders of uterus, except cervix	N85	625 (99.68%)	2 (0.32%)	627
Haemorrhage in early pregnancy	O22	584 (99.49%)	3 (0.51%)	587
Maternal care for other known or suspected fetal problems	O36	573 (98.62%)	8 (1.38%)	581
Other inflammation of vagina and vulva	N76	576 (99.31%)	4 (0.69%)	580
Total		54,469 (98.99%)	557 (1.01%)	55,026
**Complication**				
Postpartum haemorrhage	O72	771 (98.72%)	10 (1.28%)	781
Other disorders of peritoneum	K66	438 (100%)	0 (0%)	438
Other female pelvic inflammatory diseases	N73	295 (98.99%)	3 (1.01%)	298
Acute posthaemorrhagic anaemia	D62	280 (99.29%)	2 (0.71%)	282
Peritonitis	K65	184 (86.79%)	28 (13.21%)	212
Iron deficiency anaemia	D50	173 (98.86%)	2 (1.14%)	175
Premature separation of placenta with coagulation defect	O45.0	154 (99.35%)	1 (0.65%)	155
Maternal care of pelvic organs	O34	146 (100%)	0 (0%)	146
Other maternal complications	O99	138 (97.87%)	3 (2.13%)	141
Maternal care for other known or suspected fetal problems	O36	120 (99.17%)	1 (0.83%)	121
Shock, not elsewhere classified	R57	105 (95.45%)	5 (4.55%)	110
Shock following abortion and ectopic and molar pregnancy	O08.3	105 (99.06%)	1 (0.94%)	106
Other abnormal uterine and vaginal bleeding	N93	91 (100%)	0 (0%)	91
Other obstetric trauma	O71	85 (100%)	0 (0%)	85
Placenta praevia with haemorrhage	O44.1	82 (100%)	0 (0%)	82
Pre-eclampsia	O14	74 (98.67%)	1 (1.33%)	75
Other antepartum haemorrhage	O45.8	75 (100%)	0 (0%)	75
Excessive, frequent and irregular menstruation	N92	70 (100%)	0 (0%)	70
Disseminated intravascular coagulation	D65	46 (82.14%)	10 (17.86%)	56
Other complications of labour and delivery, not elsewhere classified	O75	52 (94.55%)	3 (5.45%)	55
Total		4423 (95.18%)	224 (4.82%)	4647

Table S2. Surgical procedures linked to the main diagnosis.

	Surgeries (ICD-9)											
**Main Diagnosis (ICD-10)**	65.3	65.51	65.53	65.61	65.62	66.4	66.51	66.62	68.3	68.4	68.61	Total
Ectopic pregnancy (O00)	178	2	0	12	367	3008	138	25,160	41	57	4	28,967
Intramural leiomyoma of uterus (D25.1)	38	6	1	401	25	147	336	12	4844	3005	3509	12,324
Submucous leiomyoma of uterus (D25.0)	46	5	4	233	15	98	153	0	4492	3093	2207	10,346
Benign neoplasm of ovary (D27)	857	158	121	642	502	271	93	15	802	1399	354	5214
Single delivery by caesarean section (O82)	175	0	0	3	73	51	52	0	2978	1483	7	4822
Other benign neoplasm of corpus uteri (D26.1)	16	2	47	156	21	30	19	0	2798	910	628	4627
Subserosal leiomyoma of uterus (D25.2)	19	5	3	99	7	49	141	0	1488	806	1897	4514
Other assisted single delivery (N83)	1642	49	21	198	611	561	146	102	239	116	52	3737
Other maternal complications (O99)	19	5	0	0	12	6	3061	5	129	139	0	3376
Malignant neoplasm of ovary (C56)	103	82	7	109	55	14	3	0	224	2199	50	2846
Salpingitis and oophoritis (N70)	106	23	10	360	213	848	561	57	285	93	8	2564
Maternal care of pelvic organs (O34)	7	3	0	0	0	3	1841	0	232	82	0	2168
Malignant neoplasm of endometrium (C54.1)	2	1	0	5	4	0	3	0	29	1841	273	2158
Other female pelvic inflammatory diseases (N73)	139	16	6	271	173	582	292	33	219	195	3	1929
Labour and delivery complicated by intrapartum haemorrhage, not elsewhere classified (O67)	10	0	0	7	7	1	0	0	652	484	3	1164

Table S3. Most common diagnoses with complications and comorbidities.

ICD-10 (diagnosis)	Main (N)	Complica-tion (N)	Comorbi-dity (N)
O00 (Ectopic pregancy)	28967	8	54
D25 (Leiomyoma of uterus)	27480	9	636
D25.1 (Intramur leiomyoma of uterus)	12324	4	248
D25.0 (Submucousal leiomyoma of uterus)	10346	4	235
D25.2 (Subserosal leiomyoma of uterus)	4514	1	111
D25.9 (Leiomyoma of uterus, unspecified)	296	0	42
D27 (Benign neoplasm of ovary)	5214	17	2321
O82 (Cesarean delivery without indication)	4822	10	1394
D26.1 (Other benign neoplasm of corpus uteri)	4627	3	191
N83 (Non-inflammatory disorders of ovary, fallopian tube and broad ligament)	3737	31	2790
O99 (Other maternal diseases complicating pregnancy, childbirth and the puerperium)	3376	141	4273
C56 (Malignant neoplasm of ovary)	2846	0	41
N70 (Salpingitis and oophoritis)	2564	18	2448
O34 (Maternal care for abnormality of pelvic organs)	2168	146	1329
C54.1 (Malignant neoplasm of endometrium)	2158	0	8
N73 (Other female pelvic inflammatory diseases)	1929	298	2580
O67 (Labor and delivery complicated by intrapartum hemorrhage, not elsewhere classified)	1164	31	168
N81 (Female genital prolapse)	975	0	224
C54.0 (Malignant neoplasm of isthmus uteri)	865	0	2
O45.0 (Premature separation of placenta with coagulation defect)	854	298	679
C53.0 (Malignant neoplasm of endocervix)	801	0	7
N97 (Female infertility)	794	3	165
N80 (Endometriosis)	759	0	866
O44.1 (Complete placenta previa with hemorrhage)	701	98	257
N85 (Other non-inflammatory disorders of uterus, except cervix)	653	15	627
O72 (Postpartum hemorrhage)	453	777	574
D26.0 (Other benign neoplasm of cervix uteri)	385	3	50
C53.1 (Malignant neoplasm of exocervix)	343	0	1
N71 (Inflammatory disease of uterus, except cervix)	326	7	367
O14 (Pre-eclampsia)	307	75	349
O85 (Puerperal sepsis)	259	32	27
O10 (Pre-existing hypertension complicating pregnancy, childbirth and the puerperium)	221	4	290

K65 (Peritonitis)	191	649	778
C50 (Malignant neoplasm of breast)	187	0	163
N93 (Other abnormal uterine and vaginal bleeding)	171	91	522
C54.9 (Malignant neoplasm of corpus uteri, unspecified)	155	0	5
C54.8 (Malignant neoplasm of overlapping sites of corpus uteri)	151	0	2
C18 (Malignant neoplasm of colon)	142	0	18
C54.2 Malignant neoplasm of myometrium	141	0	0
D07 (Carcinoma in situ of other and unspecified genital organs)	134	0	4
O24 (Diabetes mellitus in pregnancy, childbirth, and the puerperium)	133	0	56
